# Circulating small extracellular vesicle-encapsulated SEMA5A-IT1 attenuates myocardial ischemia–reperfusion injury after cardiac surgery with cardiopulmonary bypass

**DOI:** 10.1186/s11658-022-00395-9

**Published:** 2022-10-25

**Authors:** Ting Wu, Guoning Shi, Zhenhua Ji, Shu Wang, Lizhu Geng, Zhigang Guo

**Affiliations:** 1 Department of Cardiopulmonary Bypass, Clinical College of Chest, Tianjin Medical University, Tianjin, China; 2 Department of Cardiopulmonary Bypass, Chest Hospital, Tianjin University, Tianjin, China; 3 Department of Cardiovascular Surgery, Chest Hospital, Tianjin University, No. 261, Taierzhuang South Road, Jinnan, Tianjin, 300222 China

**Keywords:** Cardiopulmonary bypass, Cardiomyocyte injury, Small extracellular vesicles, microRNA, Apoptosis, Ferroptosis

## Abstract

Cardiomyocyte injury is a common complication during cardiac surgery with cardiopulmonary bypass (CPB). Studies have shown that circulating small extracellular vesicles (sEVs) are involved in the pathological process of cardiovascular diseases via delivering signaling molecules. This study aims to investigate the relationship between circulating sEV-encapsulated long noncoding RNAs (lncRNAs) and cardiac injury after CPB. Here, we found that the expression of sEV SEMA5A-IT1 in serum samples of patients after CPB was higher than that of pre-CPB serum samples. Moreover, serum-derived sEV SEMA5A-IT1 levels were negatively correlated with creatine kinase-MB (CK-MB) levels in patients who underwent CPB operation. Notably, circulating sEVs packaged with SEMA5A-IT1 could be uptaken by cardiomyocyte-like cells AC16 and increased SEMA5A-IT1 expression in AC16 cells. Upregulated SEMA5A-IT1 protected cardiomyocytes against hypoxia/reoxygenation injury, confirmed by increased cell viability, reduced cell apoptosis, and inhibited ferroptosis in AC16 cells. Mechanistically, SEMA5A-IT1 regulated the expression of B-cell CLL/lymphoma 2 (*BCL2*) and solute carrier family 7 member 11 (*SLC7A11*) through sponging miR-143-3p. Transfection of miR-143-3p mimics, *BCL2*, or *SLC7A11* knockdown could attenuate the protective effect of SEMA5A-IT1 on cardiomyocytes. In conclusion, we propose that SEMA5A-IT1, which is transported to cardiomyocytes through circulating sEVs, is an important regulatory molecule that protects cardiomyocytes from ischemia–reperfusion injury, providing a target for the prevention and treatment of myocardial ischemia–reperfusion injury.

## Background

Cardiac valve replacement with CPB is a common method in the clinical treatment of cardiac valve diseases [1, 2]. The success of cardiac valve replacement is closely related to the cardioprotection measures during the operation, while cardiomyocyte injury during the operation is mainly related to the stress response, hemodynamic instability, and cardiac oxygen imbalance [3, 4]. Previous studies revealed that circulating sEVs are related to the inflammatory response and acute kidney injury after CPB cardiac surgery [5, 6], suggesting that circulating sEVs have active functions. However, the effect of circulating sEVs on cardiomyocytes after CPB surgery remains unknown. Therefore, investigating the effect and molecular mechanism of circulating sEVs on cardiomyocytes after CPB surgery is of great significance for predicting the prognosis of CPB surgery and developing effective myocardial protection strategies.

Extracellular vesicles (EVs) are small lipid bilayer particles commonly released by almost all cell types [7]. Small extracellular vesicles (sEVs), a type of EV with a diameter less than 200 nm, have been identified as messengers in intercellular communication [8]. Previous studies have shown that, under different stimuli, sEVs derived from different cell types selectively package bioactive molecules (proteins, mRNAs, and noncoding RNAs) reach recipient cells through blood or other body fluids, releasing active molecules and regulating cell function [9, 10]. Researchers initially found elevated levels of microparticles (MPs) in the blood of patients undergoing cardiac surgery with CPB, and increased MPs may affect leukocytes in promoting coagulation or microvascular blockade leading to transient neurological syndromes [11, 12]. Blood-derived microparticles from patients undergoing cardiac surgery with CPB play important roles. Biró et al. [13] confirmed that MPs derived from pericardial blood of patients undergoing cardiac surgery with CPB activate the complement system by bound serum amyloid P-component and immunoglobulin M. Further studies have shown that circulating exosomes, as a special type of microparticle, play a variety of roles after CPB. Poon et al. [5] found that the abundance of exosomes was increased in post-CPB samples compared with pre-CPB samples, and increased plasma exosomes during cardiac surgery with CPB could inhibit inflammatory responses. Pat et al. [14] reported that elevated Hb-positive exosomes were found 30 min after cross-clamp release, and revealed that these plasma exosomes could lead to acute kidney injury in an animal model. However, the potential role and mechanism of circulating sEVs in cardiac function and cardiomyocytes remain unclear.

The purpose of this study was to decipher whether circulating sEVs affected the function of cardiomyocytes in patients who underwent cardiac valve replacement with CPB. Further experiments identified the differential expression of lncRNAs between sEVs before and after CPB and explored the mechanism of circulating sEVs on the function of cardiomyocytes.

## Materials and methods

### Study cohorts

We prospectively enrolled 35 patients undergoing CPB cardiac surgery from June 2020 to August 2021. This study was carried out under the Declaration of Helsinki, and the study protocol was approved by the institutional review board of Tianjin Chest Hospital (no. 2020YS-010-01).

### Isolation and identification of sEVs

In patients receiving CPB surgery (*n* = 35), whole blood was collected before and after surgery from the peripheral artery. Isolation of sEVs from serum was performed using the ExoQuick Exosome precipitation kit (catalog no. EXOQ5A-1; System Biosciences) Briefly, 4 mL of whole blood was placed in an anticoagulant tube for 2–6 h at 4 ℃. Afterward, the upper serum was transferred to a new sterile microtube and centrifuged at 3000*g* for 15 min. Then, the supernatant was transferred to a new tube, mixed with the EXO reagent, incubated for 30 min, and centrifuged. After discarding the supernatant, the isolated sEVs were resuspended and stored at −80 °C or used for further experiments. Transmission electron microscopy (TEM) and western blot detection of CD9 and CD81 were used to identify sEVs. The protein concentration of collected sEVs was determined via Micro BCA Protein Assay Kit (Thermo Fisher Scientific, MA, USA). AC16 cells were treated with pre-sEVs or post-sEVs (50 μg/mL) for indicated times.

### RNA isolation from sEVs

RNA was extracted from serum-derived sEVs using the exoRNeasy Midi Kit (Qiagen, USA). sEVs were dissolved in BB buffer (4 M guanidine hydrochloride, 2% Triton) and then lysed with TRIzol. Chloroform was added to the lysis mixture, incubated with shaking, and centrifuged to remove lipids. Following centrifugation at 12,000*g* for 15 min at 4 ℃, the upper colorless RNA-containing supernatant was transferred to a new RNase-free microtube. Isopropyl alcohol and 85% ethanol were used for RNA purification. The isolated RNAs were resuspended with DEPC H_2_O and aliquoted, and stored at −80 °C.

### Labeling and uptake of sEVs

Exosome labeling and uptake of sEVs assay were performed using ExoGlow-Membrane EV Labeling Kit (catalog no. EXOGM600A-1, SBI, USA). In brief, sEVs were incubated with a labeling dye solution for 30 min. The labeled sEVs were then cocultured with AC16 cells. The fusion efficiency was observed after incubation at 37 ℃ for 4 h, and cells were collected for follow-up experiments after 24 h.

### Western blot

Western blot was performed as described in previous studies [15]. CD9 (#60232-1-lg; 1:2000), CD81 (#66866-1-lg; 1:2000), Bax (#60267-1-lg; 1:1000), Bcl-2 (#60178-1-lg; 1:1000), β-tubulin (#66240-1-lg; 1:10,000), GAPDH (#60004-1-lg; 1:4000) monoclonal antibodies, and *SLC7A11* polyclonal antibody (#26864-1-AP; 1:2000) were obtained from Proteintech Group, Inc. (Wuhan, China).

### Real-time quantitative PCR (RT-qPCR)

The RT-qPCR experiment was carried out according to the procedures of the previous studies. TRIzol reagent (Invitrogen, USA) was used for the isolation of RNA. High-capacity cDNA reverse transcription kit (Thermo Fisher Scientific, USA) was used for cDNA synthesis. PCR reactions were performed by SYBR Green assays (TaKaRa Biotechnology, Dalian, China). U6 serves as the internal control of miRNAs, and tubulin and GAPDH serve as the internal control of lncRNAs and mRNAs. Changes in gene expression were calculated using the 2^–ΔCT^ method. The RT-qPCR primers used in this study are listed in Table 1.

**Table 1:** The sequences of primer used for RT-qPCR

Gene name	Primer sequence
NONHSAT100333	F: 5′-GTTTCTTTCATCATTTCAACTCTGGGTA-3′ R: 5′-CTGCATCCCTGTCCTGTCACTTC-3′
NONHSAT137362	F: 5′-TCTATTTCTGTGGGATCAGTGGT-3′ R: 5′-AAATCAATGAATCCAAAAGAAGG-3′
NONHSAT035383	F: 5′-CACAAAGGCACATACTAGCAGAC-3′ R: 5′-GTGTACGTATGTCTATGTGAATG-3′
NONHSAT138804	F: 5′-ACATTCCATACACTTACGCAGAC-3′ R: 5′-GACAATCTATTTCTTTGGGGACA-3′
NONHSAT095978	F: 5′-TTGCATATCAGGGTAATAATGGC-3′ R: 5′-CCTAGAAGAAATCGGTAAATTCCTAAAC-3′
Tubulin	F: 5′-GGGGAGATGTATGAAGATGATGACGA-3′ R: 5′-TGGGAGCCCTAATGAGCTGGTGA-3′
GAPDH	F: 5′-CCTGTTCGACAGTCAGCCG-3′ R: 5′-GAGAACAGTGAGCGCCTAGT-3′
miR-143-3p	F: 5′-CTGGCGTTGAGATGAAGCAC-3′ R: 5′-CAGAGCAGGGTCCGAGGTA-3′
U6	F: 5′-CTCGCTTCGGCAGCACA-3′ R: 5′-AACGCTTCACGAATTTGCGT-3′

*F* forward, *R* reverse

### Cell culture and transfection

Human cardiomyocyte cell line AC16 was purchased from the Bena Culture Collection (BNCC339980, BNCC, Beijing, China), and cultured in DMEM-H medium (BNCC, Beijing, China) at 37 ℃, 5% CO_2_. AC16 cell line was derived from the fusion of primary cells from adult human ventricular heart tissues with SV40 transformed, uridine auxotroph human fibroblasts, devoid of mitochondrial DNA [16]. The cells can be used to study cardiac gene expression and function, during normal development and under pathological conditions at cellular, organellar, and molecular levels.

For SEMA5A-IT1 overexpression, the lentiviral vectors of SEMA5A-IT1 (SEMA5A-IT1_OE) or negative vectors were used to infect AC16 cells. For cell transfection, Lipofectamine 3000 (Invitrogen, USA) was used according to the manufacturer’s instructions. The lentiviral vectors of SEMA5A-IT1, miR-143-3p mimics, and small interfering RNAs (siRNAs) targeting *BCL2* (si-BCL2) or *SLC7A11* (si-SLC7A11) were all purchased from Oligobiobio Co., Ltd. (Beijing, China), and the corresponding sequences are listed in Table 2.

**Table 2:** The sequences of siRNAs

siRNA	Sequence (5′ → 3′)
si-*BCL2*	Sense: GGUACGAUAACCGGGAGAUAGUGAU Antisense: AUCACUAUCUCCCGGUUAUCGUACC
si-*SLC7A11*	Sense: GAGUCUGGGUGGAACUCCUCAUAAU Antisense: AUUAUGAGGAGUUCCACCCAGACUC

### Establishment of hypoxia/reoxygenation (H/R) model

AC16 cells were first cultured to 80% confluence in a complete CM1-1 medium. Afterward, the cells were cultured in serum-free and sugar-free medium and placed in anoxic incubators containing 95% N_2_ and 5% CO_2_. After 6 h of hypoxia, the medium was replaced with fresh complete CM1-1 medium in 5% CO_2_.

### Microarray and computational analysis

Total RNA was transcribed in vitro to synthesize cRNA. After purification and quantification of cRNA, the cRNA was diluted to 625 ng/μL for subsequent experiments. After purifying the second-cycle sing-reactive cDNA, 5.5 μg single-stranded cDNA (sscDNA) was diluted to 31.2 μL in enzyme-free water for subsequent fragmentation and labeling. The segmented labeled samples were added to the chip of the corresponding model and put into a GeneChip Hybridization Oven 645, and chip hybridization was carried out at a specific temperature and rotating speed. After reaching the specified time, GeneChip Fluidics Station 450 was used for washing and staining according to the corresponding protocol. After completion, GeneChip 3000 7G scanner was used for scanning. The scanner captures the fluorescence signal and converts the signal through GCOS software to obtain the signal value of each probe and generate CEL files.

### Dual-luciferase reporter assay

The sequences of SEMA5A-IT1, *BCL2*, or *SLC7A11* possessing the wild-type miR-143-3p binding sites were predicted by using bioinformatics analysis. The luciferase plasmids containing wild-type (wt) or mutated (mut) miR-143-3p binding sites were provided by Genema (Shanghai, China). These plasmids were transiently transfected into HEK293T cells with miR-143-3p mimic or negative control (NC) mimic. Luciferase activity was measured after 48 h of transfection.

### RNA immunoprecipitation (RIP) assay

RIP was performed by Magna RIP RNA Binding Protein Immunoprecipitation Kit (Millipore, Billerica, MA). anti-AGO2 antibody or IgG was bound to magnetic beads and then incubated with AC16 cell lysates by RIP lysis buffer. The precipitated RNA was then isolated, and the expression of SEMA5A-IT1 and miR-143-3p was detected using RT-qPCR.

### Lipid peroxidation assay

AC16 cells were lysed with cell lysis buffer, and the supernatant was prepared with thiobarbituric acid (TBA)–glacial acetic acid reagent. After incubation at 95 °C for 1 h, the malondialdehyde (MDA)–TBA adduct was quantified by spectrophotometry at 532 nm.

### Species detection of reactive oxygen species (ROS)

After indicated treatment, AC16 cells were digested by trypsin and suspended in a culture medium. Cells were supplemented with 10 μM C 11-Bodipy solution (Thermo Fisher, USA) and incubated in a dark incubator for 30 min. Cells were washed twice with PBS. Fluorescence of C11-Bodipy was observed by fluorescence microscope (Nikon, Japan).

### Statistical analysis

Continuous variables are presented as median and interquartile ranges as appropriate. The normal distribution of continuous variables was evaluated with Shapiro–Wilk test. Student’s *t*-test (two-sided) or analysis of variance was used for two or more group comparisons. Data were analyzed using GraphPad Prism 8.0 software (GraphPad, San Diego, CA), expressed as mean ± standard error of the mean (SEM). All the experiments were performed as three independent experiments in triplicate. *p* < 0.05 indicated statistical differences.

## Results

### Circulating sEV SEMA5A-IT1 was significantly elevated after CPB surgery

**Fig. 1:**
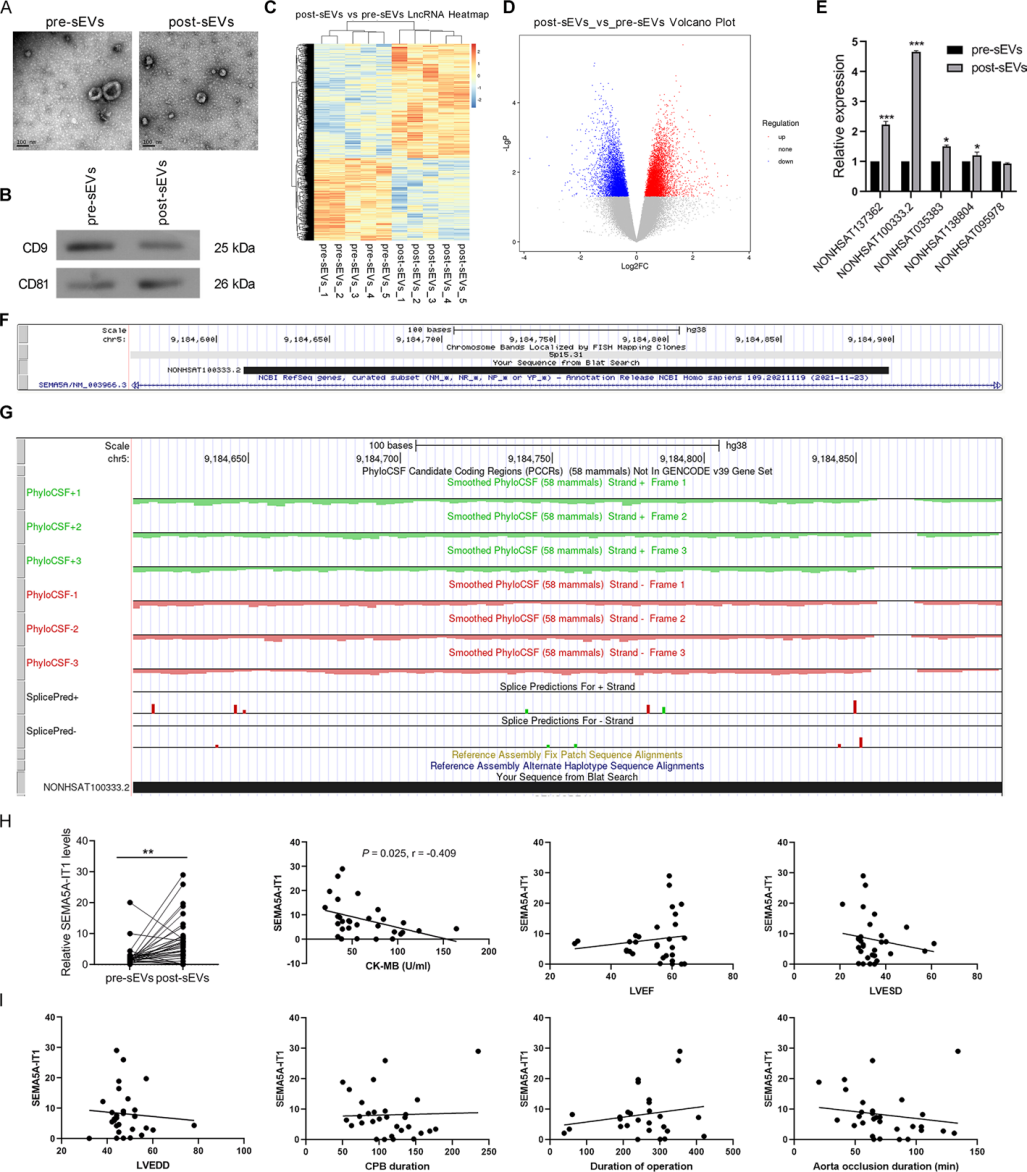
Circulating sEV SEMA5A-IT1 was significantly elevated after CPB surgery. **A** Representative picture of the ultrastructure of pre-sEVs and post-sEVs observed by TEM. **B** The protein levels of CD9 and CD81 in the two types of sEV. **C** Hierarchical clustering analysis of differentially expressed lncRNAs in pre-sEVs (*n* = 5) and post-sEVs (*n* = 5) using lncRNA microarray. **D** Volcano plot of differentially expressed lncRNAs in pre-sEVs and post-sEVs. The red dots indicate upregulated lncRNAs in post-sEVs. Green dots indicate downregulated lncRNAs in post-sEVs. **E** Validation of the five lncRNAs with the most significant difference by qRT-PCR. **F** The location of SEMA5A-IT1 on the human chromosome from UCSC Genome Browser (https://genome.ucsc.edu/). **G** The coding-potential analysis using PhyloCSF. *p < 0.05. ***p < 0.001. **H** RT-qPCR was performed to examine the levels of SEMA5A-IT1 in sEVs derived from patients’ serum before and after CPB. **I** The correlation between SEMA5A-IT1 in post-EVs and CK-MB, LVEF, LVESD, LVEDD, CPB duration, operation duration, and aorta occlusion duration. **p < 0.01

We isolated serum-derived sEVs from five patients before and after cardiac surgery with CPB. We defined sEVs before CPB as pre-sEVs and those after CPB as post-sEVs. TEM analysis showed that the purified sEVs exhibited cup- or sphere-shaped morphology with a diameter of 40–150 nm (Fig. 1A). Further, western blotting analysis verified that these particles positively expressed CD9 and CD81, which are sEV surface markers (Fig. 1B). Together, these results confirmed that the isolated circulating nanoparticles were sEVs.

To identify sEV lncRNA expression profiles in serum samples of patients after CPB, total RNAs isolated from sEVs were subjected to microarray analysis. By applying a paired two-tailed *t*-test, microarray assays showed that sEV lncRNAs were expressed differentially in serum before and after CPB (Fig. 1C). As displayed in Fig. 1D, a total of 2133 lncRNAs were significantly altered in the two considered groups (fold change ≥ 2.0 and *P* < 0.05). Out of the 2133 altered lncRNAs, 905 were upregulated in the samples after CPB compared with samples before CPB, while 1228 lncRNAs were downregulated. Five lncRNAs with the most significant difference (NONHSAT137362, NONHSAT100333.2, NONHSAT035383, NONHSAT138804, and NONHSAT095978) were verified by qRT-PCR. As shown in Fig. 1E, the expression of NONHSAT100333.2 was significantly upregulated in the post-sEV group. NONHSAT100333.2 is a 286 nt lncRNA transcript from the 11th intron of the *SEMA5A* gene (Fig. 1F), so we named the lncRNA SEMA5A-IT1. In addition, the coding-potential analysis using PhyloCSF showed that all PhyloCSF scores were negative (Fig. 1G), providing evidence for the noncoding nature of SEMA5A-IT1.

### Correlations between the levels of SEMA5A-IT1 in circulating sEVs and the clinical course

We analyzed the amount of SEMA5A-IT1 in preoperative and postoperative sEVs in a separate cohort of 30 patients, and the clinical characteristics of these patients are presented in Table 3. The expression of SEMA5A-IT1 was significantly increased in post-sEV samples compared with pre-sEV samples (Fig. 1H). Further, we analyzed correlations between SEMA5A-IT1 levels in serum sEVs after CPB and the clinical course. As shown in Fig. 1I, there was a negative association between postoperative sEVs SEMA5A-IT1 concentrations and postoperative CK-MB, the marker for cardiomyocyte damage. However, there were no significant correlations between SEMA5A-IT1 concentration and CPB duration, operation duration, aorta occlusion duration, postoperative left ventricular ejection fraction (LVEF), left ventricular end-systolic dimension (LVESD), or left ventricular end-diastolic dimension (LVEDD).

**Table 3:** Main demographic and clinical characteristics of 30 patients

Demographic characteristics	
Number of patients	30
Average age, years (range)	55.4 (30–77)
Sex ratio (M/F)	1.0 (15/15)
Surgical characteristics	
Type of surgery:	
ASD	2 (6.7%)
AVR	5 (16.6%)
DVR	2 (6.7%)
MVR	21 (70.0%)
Duration of surgery (min)	250 (38–420)
Duration of CPB (min)	113 (50–235)

*M/F* male/female, *ASD* atrial septal defect, *AVR* aortic valve replacement, *DVR* double valve replacement, *MVR* mitral valve replacement, *CPB* cardiopulmonary bypass

### SEMA5A-IT1 shuttled in post-sEVs protects against myocardial H/R injury

**Fig. 2:**
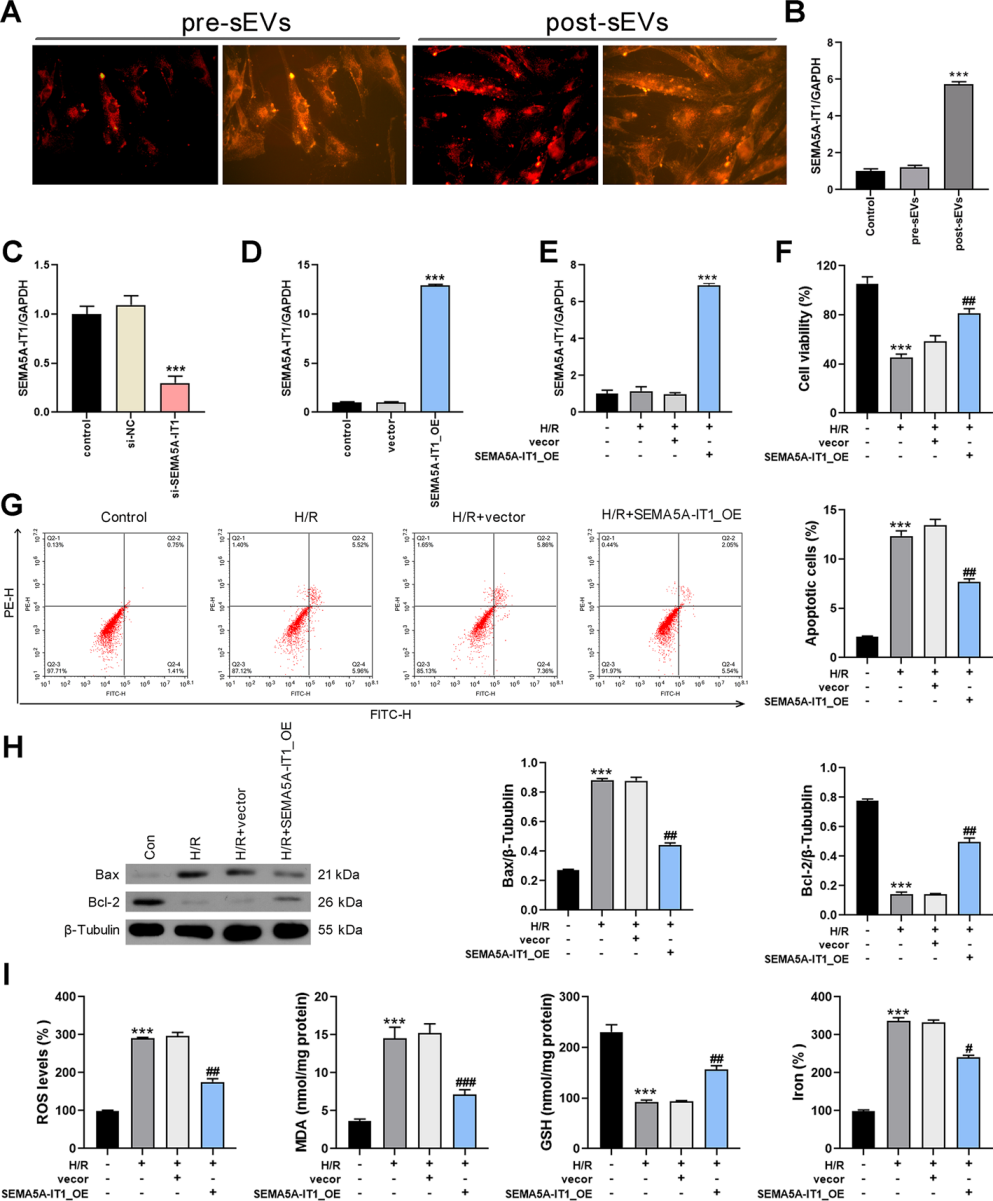
SEMA5A-IT1 shuttled in post-sEVs protects against myocardial H/R injury. **A** The uptake of sEVs by human AC16 cells. **B** RT-qPCR was performed to examine the expression of SEMA5A-IT1 in AC16 cells co-cultured with pre-sEVs or post-sEVs. **C** The expression of SEMA5A-IT1 in AC16 cells transfected with si-SEMA5A-IT1. **D** The expression of SEMA5A-IT1 in AC16 cells infected with SEMA5A-IT1-overexpressing lentiviral vectors. **E** The expression of SEMA5A-IT1 in AC16 cells infected with SEMA5A-IT1-overexpressing plasmids and exposure to H/R damage. **F** Cell viability was detected in different groups by CCK8 assay. **G** Cell apoptosis was detected in different groups by flow cytometry of Annexin V/IP staining. **H** Western blot was used to detect Bcl-2 and Bax protein expression. ***p < 0.001 compared with the control group. **I** ROS, MDA, GSH, and iron accumulation were detected in each group. #*p* < 0.05, ##*p* < 0.01, ###*p* < 0.001 compared with the SEMA5A-IT1_OE group

Since high levels of SEMA5A-IT1 in post-sEVs are associated with favorable cardiac function outcomes, we wondered whether SEMA5A-IT1 in post-sEVs protects against myocardial ischemia–reperfusion injury. First, these sEVs were labeled with red fluorescent membrane dye and co-cultured with human AC16 cells. After being incubated for 24 h, these sEVs were internalized by AC16 cells, as observed using fluorescence microscopy (Fig. 2A). Further, compared with pre-EVs, post-EVs significantly promoted the expression of SEMA5A-IT1 in AC16 cells (Fig. 2B). Further, knockdown or overexpression of SEMA5A-IT1 was performed in AC16 cells, and the transfection validity was confirmed by qRT-PCR (Fig. 2C, D). Next, we established an H/R cell model to evaluate the role of SEMA5A-IT1 in H/R-induced myocardial injury. As shown in Fig. 2E, transfection with SEMA5A-IT1-overexpressing plasmids significantly increased SEMA5A-IT1 expression in AC16 cells exposed to H/R treatment. The CCK8 assay showed that SEMA5A-IT1 overexpression protected cardiomyocytes against H/R injury (Fig. 2F). The flow cytometry results indicated that the H/R group increased the percentage of apoptotic cells, whereas SEMA5A-IT1 elevation reduced H/R-mediated apoptosis (Fig. 2G). Meanwhile, western blot results indicated that SEMA5A-IT1 overexpression reversed the expression of Bax and Bcl-2 in AC16 cells induced by H/R treatment (Fig. 2H). On the basis of the results, we concluded that SEMA5A-IT1 shuttled in post-EVs can be captured by cardiomyocytes and protect against myocardial ischemia–reperfusion injury.

Recent studies revealed that ferroptosis exerts a crucial role in the pathophysiology of cardiovascular disease, including ischemia/reperfusion [17, 18]. Therefore, we examined the effect of SEMA5A-IT1 on ferroptosis. As shown in Fig. 2I, the results demonstrated that SEMA5A-IT1 overexpression increased GSH, while decreasing reactive oxygen species (ROS) production, MDA, and iron accumulation in H/R-treated AC16 cells. The above results indicated that SEMA5A-IT1 encapsulated in sEVs inhibited H/R-induced myocardial cell death via reduction of apoptosis and ferroptosis.

### SEMA5A-IT1 sponges miR-143-3p

**Fig. 3:**
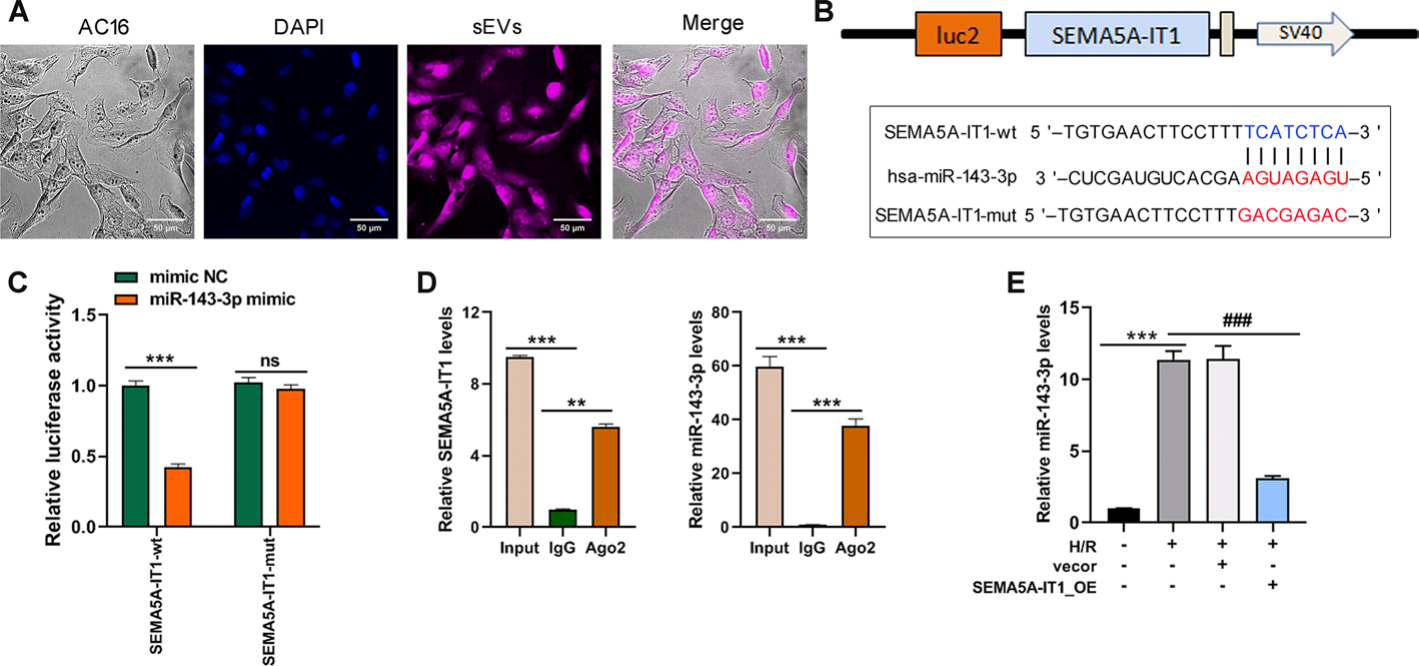
SEMA5A-IT1 sponges miR-143-3p and negatively regulates the expression of miR-143-3p. **A** AC16 cells were incubated with labeled sEVs for 4 h and then stained with DAPI. The uptake of sEVs was observed under a fluorescence microscope. Scale bars, 50 μm. **B** The putative miR-143-3p bound sequence of the wild-type (wt) and mutant (mut) sequence of SEMA5A-IT1 from the miRDB database. **C** Luciferase reporter assay was conducted to verify the binding relationship between SEMA5A-IT1 and miR-143-3p. **D** The Ago2-RIP assay was conducted to reveal that both endogenous SEMA5A-IT1 and miR-143-3p could be pulled down by the AGO2 antibody. **E** RT-qPCR was used to detect the expression of miR-143-3p in AC16 cells after being infected with SEMA5A-IT1-overexpressing plasmids and exposure to H/R damage. SEMA5A-IT1-wt, SEMA5A-IT1 wild type; SEMA5A-IT1-mut, mutant SEMA5A-IT1. *p < 0.05, **p < 0.01, ***p < 0.001, and ns indicates no significant difference

To explore the mechanism by which SEMA5A-IT1 induces cell injury in cardiomyocytes, we first detected its subcellular localization in AC16 cells by fluorescence in situ hybridization, it was found that SEMA5A-IT1 was distributed predominantly in the cell cytoplasm (Fig. 3A). Thus, we hypothesized that SEMA5A-IT1 might act as a miRNA sponge to prevent miRNAs from binding with their target mRNAs. Through the miRDB database (http://mirdb.org/custom.html), miR-143-3p was identified as the potential target of SEMA5A-IT1 (Fig. 3B). Previous studies revealed that miR-143-3p plays critical regulatory roles in various pathophysiological processes in heart diseases [19–21]. Further, luciferase plasmids containing wild-type and mutant SEMA5A-IT1 were constructed and co-transfected with miR-143-3p mimic into HEK293 cells. The luciferase reporter assay verified that miR-143-3p mimic significantly reduced the luciferase activity of the pmirGLO-SEMA5A-IT1-WT vector but failed to decrease that of the mutant vector (Fig. 3C). Moreover, the Ago2-RIP assay revealed that both endogenous SEMA5A-IT1 and miR-143-3p could be pulled down by the AGO2 antibody (Fig. 3D), further validating their binding potential. In addition, qRT-PCR revealed that H/R treatment promoted miR-143-3p expression, whereas overexpression of SEMA5A-IT1 repressed miR-143-3p expression (Fig. 3E). These data support that SEMA5A-IT1 directly targets and inhibits miR-143-3p expression.

### miR-143-3p negatively regulates *BCL2* and *SLC7A11* expression

**Fig. 4:**
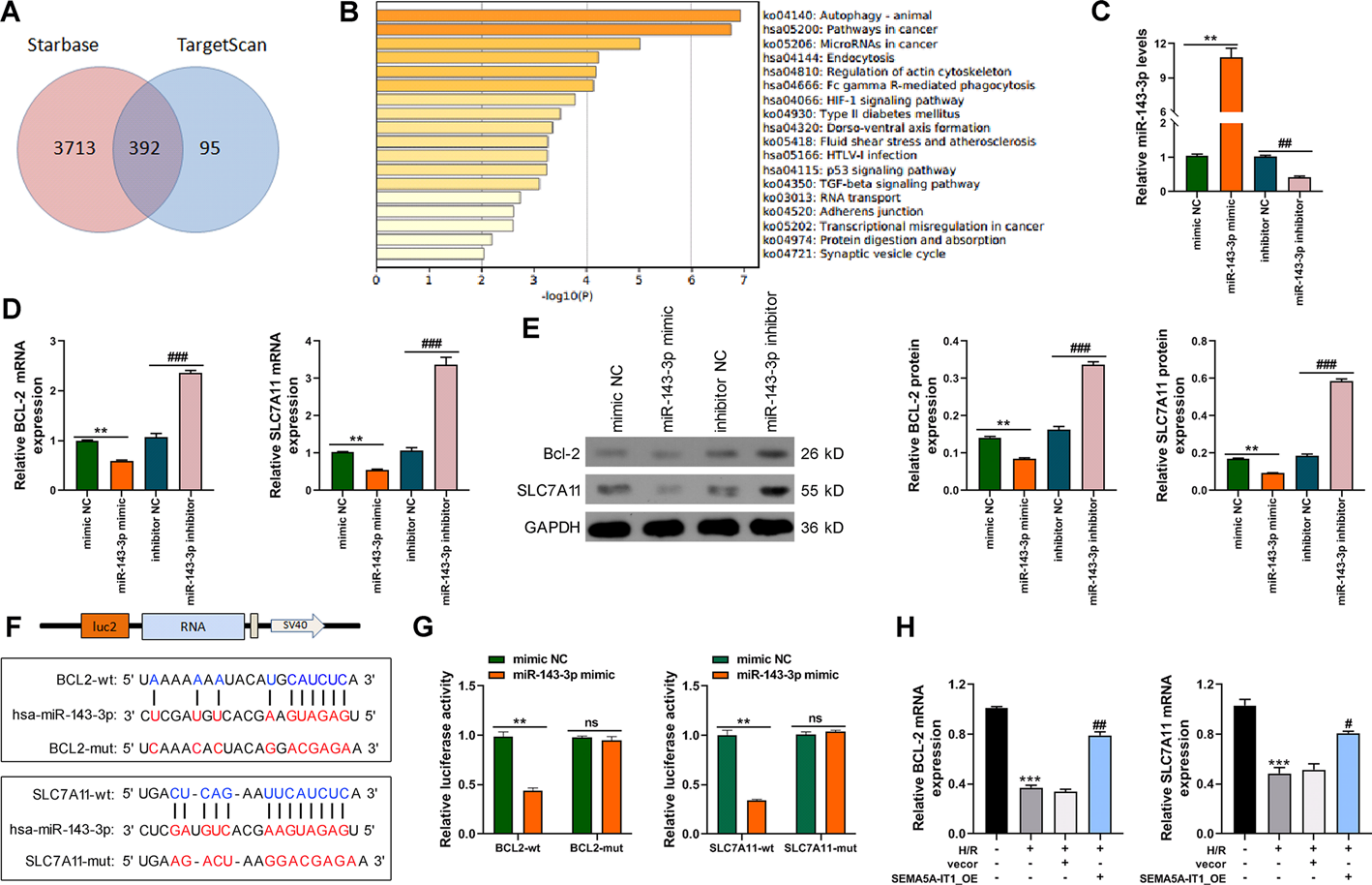
miR-143-3p negatively regulates *BCL2* and *SLC7A11* expression. **A** TargetScan 7.2 and Starbase 3.0 were used to predict the potential targets of miR-143-3p. **B** KEGG enrichment of the 392 genes by Metascape server. **C** RT-qPCR was performed to detect the expression of miR-143-3p in AC16 cells transfected with miR-143-3p mimic or inhibitor. **D**, **E** RT-qPCR (**D**) and western blot (**E**) were performed to detect the levels of *BCL2* and *SLC7A11* in AC16 cells transfected with miR-143-3p mimic or inhibitor. **F** The putative miR-143-3p bound sequence of the wild type (wt) and mutant (mut) sequence of *BCL2* or *SLC7A11* from the Starbase 3.0 database. **G** The dual-luciferase assays were performed to verify the binding relationship between *BCL2* or *SLC7A11* and miR-143-3p. **H** RT-qPCR was performed to measure the levels of *BCL2* and *SLC7A11* in AC16 cells infected with SEMA5A-IT1-overexpressing plasmids and exposure to H/R damage. **p < 0.01, ***p < 0.001 compared with the control group. #*p* < 0.05, ##*p* < 0.01, ###*p* < 0.001 compared with the SEMA5A-IT1_OE group

Next, TargetScan 7.2 and Starbase 3.0 were used to predict the potential targets of miR-143-3p, and 392 genes were predicted by both databases (Fig. 4A). The 392 genes were mainly enriched in autophagy and pathways in cancer (Fig. 4B), of which *BCL2* and *SLC7A11*, which are enriched in the cellular response to oxidative stress, attracted our interest. Then, to clarify the relationship between miR-143-3p and *BCL2* or *SLC7A11*, miR-143-3p mimic or inhibitor was transfected into AC16 cells to increase or decrease the expression of miR-143-3p (Fig. 4C). RT-qPCR results revealed that miR-143-3p mimic led to reduced levels of *BCL2* and *SLC7A11*, while miR-143-3p inhibitor caused contrary results in AC16 cells (Fig. 4D), which was also confirmed by western blot (Fig. 4E). Moreover, luciferase plasmids containing wild-type and mutant *BCL2* or *SLC7A11* were constructed on the basis of bioinformatics predictions of binding sites (Fig. 4F). The dual-luciferase reporter gene assays demonstrated that luciferase activity of wt-*BCL2* or wt-*SLC7A11* was inhibited in the presence of miR-143-3p mimic, while no evident differences were found in mut-*BCL2* or mut-*SLC7A11* (Fig. 4G). Besides, qRT-PCR revealed that H/R treatment decreased the expression of *BCL2* and *SLC7A11*, while SEMA5A-IT1 overexpression reversed the inhibitory effect of H/R on *BCL2* and *SLC7A11* expression (Fig. 4H).

### SEMA5A-IT1 protects cardiomyocytes from H/R damage by upregulating *BCL2* and *SLC7A11*

**Fig. 5:**
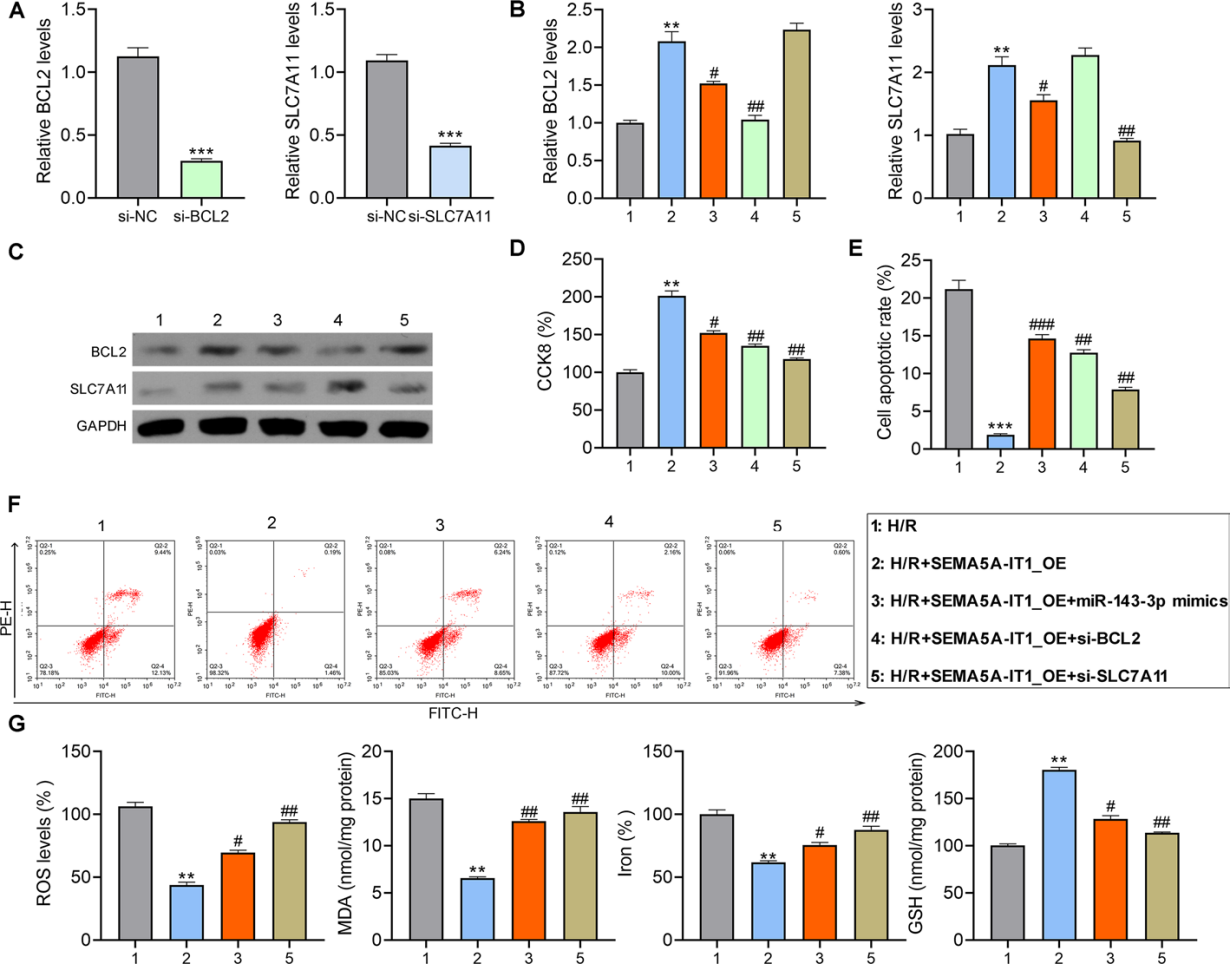
SEMA5A-IT1 protects cardiomyocytes from H/R damage by upregulating *BCL2* and *SLC7A11*. **A** RT-qPCR was performed to measure the levels of *BCL2* and *SLC7A11* in AC16 cells transfected with siRNAs targeting *BCL2* or *SLC7A11*. Next, after AC16 cells were co-transfected with si-*BCL2*, si-*SLC7A11*, or miR-143-3p mimic and SEMA5A-IT1-overexpressing plasmids, they were exposed to H/R condition. **B**–**G** RT-qPCR to measure the levels of *BCL2* and *SLC7A11* mRNA (**B**), western blot to assess the levels of *BCL2* and *SLC7A11* protein (**C**), CCK8 assay for cell viability (**D**), flow cytometry for cell apoptosis (**E**, **F**), and detection of ROS, MDA, GSH, and iron accumulation (**G**). **p < 0.01, ***p < 0.001 compared with the H/R group. #*p* < 0.05, ##*p* < 0.01, ###*p* < 0.001 compared with the SEMA5A-IT1_OE group

The siRNAs targeting *BCL2* or *SLC7A11* were transfected into AC16 cells, and the downregulation of *BCL2* and *SLC7A11* was confirmed (Fig. 5A). Next, AC16 cells were co-transfected into the *BCL2*, *SLC7A11* siRNAs, or miR-143-3p mimic with SEMA5A-IT1-overexpressing plasmids, and then exposed to H/R condition. RT-qPCR and western blot showed that SEMA5A-IT1 overexpression elevated the expression of *BCL2* and *SLC7A11*, which could be repressed by miR-143-3p mimic or their corresponding siRNA in AC16 cells, revealed by RT-PCR and western blot (Fig. 5B, C). The results of the CCK8 assay and flow cytometry revealed that, compared with H/R groups, cell proliferation of AC16 was significantly enhanced, and cell apoptosis was inhibited in the SEMA5A-IT1_OE group. However, when *BCL2* or *SLC7A11* was knocked down or miR-143-3p mimic was transfected in AC16 cells, this protective effect was found to be weakened (Fig. 5D–F). In addition, *SLC7A11* is a crucial negative regulator of ferroptosis [22], and we wonder whether it is a key participant in SEMA5A-IT1-mediated ferroptosis. We detected ferroptosis-related indicators, ROS, lipid peroxidation, and iron accumulation. As shown in Fig. 5G, the overexpression of SEMA5A-IT1 significantly inhibited H/R-induced ROS production, MDA levels, and iron accumulation, and promoted the activity of GSH, while these effects were eliminated by transfection of si-*SLC7A11* or miR-143-3p mimics.

## Discussion

In this study, we found that SEMA5A-IT1 expression in serum-derived sEVs of patients after CPB was significantly higher than that before CPB and was negatively correlated with the content of CK-MB in serum after CPB. Co-culture of AC16 cells with post-sEVs increased SEMA5A-IT1 expression. Moreover, overexpression of SEMA5A-IT1 could protect cardiomyocytes from H/R injury. Mechanistic studies revealed that SEMA5A-IT1 overexpression upregulated the expression of *BCL2* and *SLC7A11* through sponging miR-143-3p, thereby protecting cardiomyocytes against apoptotic and ferroptosis cell death. Here, we revealed a novel association between circulating sEVs and cardiomyocytes, providing a new target for alleviating myocardial injury during CPB operation.

Previous studies have suggested that circulating sEVs from patients undergoing CPB influence inflammatory responses and acute kidney injury (AKI) [23, 24]. Since sEVs can act on different types of cells, we speculated whether circulating sEVs could also influence the function of cardiomyocytes. As expected, by co-culturing sEVs with human cardiomyocytes, we found that sEVs could be internalized by cardiomyocytes. Cellular functional studies have shown that post-CPB serum-derived sEVs protected cardiomyocytes from H/R-induced apoptosis and ferroptosis. These data suggest that circulating sEVs can influence cardiomyocyte function after CPB.

Circulating sEVs may serve as a potential noninvasive biomarker for patient prognosis after cardiac surgery with CPB [25]. Li et al. [6] found that the concentration of circulating microparticles in the acute heart failure (AHF) group at 12 h after surgery was higher than that in the non-AHF group. Further logistic regression analysis indicated that the concentration of microparticles at 12 h after surgery was an independent risk factor for AHF. Ma et al. [26] revealed that the concentration of endothelial microparticles could predict CPB-related acute kidney injury at 12 h and 3 days post-CPB with high specificities. EVs mediate cell-to-cell communication by delivering biologically active molecules, including noncoding RNAs, mRNAs, and proteins. A growing number of studies have shown that sEV lncRNAs are involved in the dynamic evolution of cardiovascular diseases via various pathways, involving various aspects of pathophysiology. Studies have confirmed that lncRNAs are involved in the pathological progression of atherosclerosis, acute cardiomyocyte infarction and ischemia–reperfusion injury, cardiac angiogenesis, repair, and protection against cardiac aging [24, 27, 28]. Differential changes of lncRNAs in patient serum sEVs make them potential biomarkers for the diagnosis and treatment of cardiovascular diseases [29, 30]. In this study, we investigated the lncRNA transcriptome in pre-sEVs and post-sEVs and found that SEMA5A-IT1 was significantly elevated in post-sEVs. Further analysis showed that elevated SEMA5A-IT1 in post-sEVs was associated with a good prognosis after CPB, suggesting that sEVs SEMA5A-IT1 may be a potential biomarker for the prognosis of patients after cardiac surgery with CPB.

LncRNAs play multiple functions in cardiac development and diseases [31–33]. The subcellular localization of lncRNA is a key factor determining its function. Cytoplasmic lncRNAs mainly regulate mRNA stability, mRNA translation, miRNA processing, and function, while lncRNAs located in the nucleus epigenetically regulate chromatin remodeling, structure, and transcription [34]. Cytoplasmic lncRNAs usually act as competing endogenous RNAs (ceRNA) by binding miRNAs [35]. In the present study, we observed that SEMA5A-IT1 was mainly localized in the cytoplasm. Further experiments showed that SEMA5A-IT1 could bind miR-143-3p and repress its expression, leading to the upregulation of its targets, *BCL2* and *SLC7A11*. As SEMA5A-IT1 and miR-143-3p are all associated with Ago2, the core component of the RNA-induced silencing complex (RISC), the regulation between SEMA5A-IT1 and miR-143-3p might be similar to the miRNA-mediated silencing of protein-coding genes. Rescue experiments further demonstrated that SEMA5A-IT1 facilitated cell viability of cardiomyocytes through reduction of apoptosis and ferroptosis by binding with miR-143-3p to upregulate *BCL2* and *SLC7A11*. *BCL2* is an integral outer mitochondrial membrane protein that can prevent apoptotic cell death [36]. *SLC7A11* is a member of a heteromeric, sodium-independent, anionic amino acid transport system that is highly specific for cysteine and glutamate. *SLC7A11* has been identified as a negative regulator of ferroptosis, and has been shown to transport cysteine into cells in exchange for intracellular glutamate facilitating glutathione synthesis and reduce ROS-mediated stress [37]. Ferroptosis is a new type of iron-dependent cell death characterized by excessive lipid peroxidation and intracellular iron accumulation. Recently, a growing number of epidemiological studies and animal experiments have shown that ferroptosis plays an important character in the pathophysiology of cardiovascular disease, including ischemia/reperfusion injury [38]. In this study, we found that overexpression of SEMA5A-IT1 significantly inhibited H/R-mediated ferroptosis, while *SLC7A11* deficiency attenuated this protective effect, suggesting that SEMA5A-IT1 is involved in the regulation of ferroptosis through *SLC7A11*.

In this study, we confirmed the protective effect of lncRNA SEMA5A-IT1 carried by sEVs after CPB on cardiomyocytes after ischemia–reperfusion, which provides reference for the clinical application of sEV-based treatment strategies. However, there are still many questions to be resolved before sEV-based SEMA5A-IT1 can be considered as a realistic therapeutic approach for cardioprotection. It is well known that sEVs are secreted by activated cells into the circulation and act as mediators of intercellular communication. Here, we have demonstrated the protective effect of sEVs SEMA5A-IT1 in circulating on cardiomyocytes against ischemia–reperfusion injury, but the cellular origin of these sEVs remains uncertain. It has been reported that most of these EVs after CPB surgery originate from erythrocytes and platelets but are also produced by other cells such as leukocytes, monocytes, macrophages, and endotheliocytes [39, 40]. Platelet-derived EVs are the most abundant among all types of EV in the circulation, and previous studies have demonstrated that platelets release a significantly increased number of EVs after CPB [5]. Thus, we propose that platelets could be one of the potential origins of sEV SEMA5A-IT1. Furthermore, we cannot exclude the possibility that complications, medication, and other factors may also affect the abundance of SEMA5A-IT1 after CPB, which also needs to be further investigated. In addition, previous studies showed that circulating EV cardiac surgery also affected renal function [26, 41]. Our study does not explore the effects of these EVs on renal function, and these questions must be addressed in future studies.

## Conclusions

We demonstrated that circulating sEVs could deliver SEMA5A-IT1 to protect cardiomyocytes against apoptosis and ferroptosis via sponging miR-143-3p to upregulate *BCL2* and *SLC7A11* levels. Our data suggest that circulating SEMA5A-IT1 may be a potential biomarker and a therapeutic target for cardiomyocyte damage.

## Data Availability

The datasets supporting the conclusions of this article are included within the article and its additional files.

## Abbreviations

CPB: Cardiopulmonary bypass
lncRNAs: Long noncoding RNAs
CK-MB: Creatine kinase-MB
BCL2: B-cell CLL/lymphoma 2
SLC7A11: Solute carrier family 7 member 11
MPs: Microparticles
TEM: Transmission electron microscopy
H/R: Hypoxia/reoxygenation
RT-qPCR: Real-time quantitative PCR
ROS: Reactive oxygen species
TBA: Thiobarbituric acid
MDA: Malondialdehyde
SEM: Standard error of the mean
LVEF: Left ventricular ejection fraction
LVESD: Left ventricular end-systolic dimension
LVEDD: Left ventricular end-diastolic dimension
AKI: Acute kidney injury
